# Complete Genome Sequence of *Bacillus megaterium* Siphophage Silence

**DOI:** 10.1128/genomeA.01130-15

**Published:** 2015-10-08

**Authors:** Jonathan A. Solis, Nicholas G. Farmer, Jesse L. Cahill, Eric S. Rasche, Gabriel F. Kuty Everett

**Affiliations:** Center for Phage Technology, Texas A&M University, College Station, Texas, USA

## Abstract

Silence is a newly isolated siphophage that infects *Bacillus megaterium*, a soil bacterium that is used readily in research and commercial applications. A study of *B. megaterium* phage Silence will enhance our knowledge of the diversity of *Bacillus* phages. Here, we describe the complete genome sequence and annotated features of Silence.

## GENOME ANNOUNCEMENT

*Bacillus megaterium* is a Gram-positive, saprophytic, spore-forming bacterium whose large cell size makes it an ideal model for research in cell morphology. It is also used commercially as a host for the production of vitamin B_12_, penicillin acylase, and amylases (1). *B. megaterium* bacteriophages may be a valuable tool to advance the use of this bacterium. Here, we present the genome sequence of a newly isolated *B. megaterium* siphophage, Silence, which was isolated on the asporogenic *B. megaterium* strain KM (ATCC 13632).

Bacteriophage Silence was isolated from a soil sample collected in College Station, TX. Phage DNA was sequenced in an Illumina MiSeq 250-bp paired-end run with a 550-bp insert library at the Genomic Sequencing and Analysis Facility at the University of Texas (Austin, TX). Quality-controlled trimmed reads were assembled to a single contig of circular assembly at 34.4× coverage using SPAdes version 3.5.0 (2). The contig was confirmed to be complete by PCR using primers that face the upstream and downstream ends of the contig. Products from the PCR amplification of the junctions of concatemeric molecules were sequenced by Sanger sequencing (Eton Bioscience, San Diego, CA). Genes were predicted using GeneMarkS (3) and corrected using software tools available on the Center for Phage Technology (CPT) Galaxy instance (https://cpt.tamu.edu/galaxy-public/). The morphology of Silence was determined using transmission electron microscopy performed at the Texas A&M University Microscopy and Imaging Center.

Silence contains a 40,001-bp double-stranded DNA (dsDNA) genome, with a G+C content of 38.3% and coding density of 94.3%. It contains 66 coding sequences, 33 of which have annotated functions, as determined by InterProScan and BLASTp searches (4, 5). Silence was also found to infect the plasmidless *B. megaterium* QM B1551 derivative PV361 (6). BLASTn analysis shows that Silence has no similarity to any phages in the current GenBank database (7). Silence is predicted to use a *pac*-type DNA packaging mechanism based on TerL homology with the TerL proteins of phages with known packaging strategies; accordingly, it was opened to the *terS* gene for annotation purposes (8).

Genes related to DNA replication and recombination activities were identified, including a single-stranded DNA (ssDNA)-annealing protein, dsDNA-binding proteins, DNA polymerase, helicase, and Holliday junction resolvase. The annotated genes encoding morphogenesis proteins were those for the prohead protease, major capsid protein, tape-measure protein (and related chaperones), major tail protein, and tailspike proteins. Genes whose products are involved in DNA packaging were also identified, including the small terminase, large terminase, and portal protein. Interestingly, the large terminase subunit sequence is interrupted by a group I intron encoding a homing endonuclease. A dUTPase involved in maintaining cellular dUMP levels was also identified (9).

Silence encodes an FtsK/SpoIIIE-like protein. SpoIIIE is essential for sporulation and functions as a DNA translocase that moves the chromosome across the sporulation septum into the forespore (10). The function of this protein in the phage life cycle has yet to be determined.

### Nucleotide sequence accession number.

The genome sequence of Silence was contributed as accession no. KT001912 to GenBank.
